# Supplementary material for: A Novel Spo11 Homologue Functions as a Positive Regulator in Cyst Differentiation in Giardia lamblia
Source: Int J Mol Sci. 2021 Nov 2;22(21):11902. doi: 10.3390/ijms222111902 (PMC8584520; doi:10.3390/ijms222111902)
Supplement: Supplementary file 1 [file ijms-22-11902-s001.zip › S1 to S9 and Table S2.pdf]

Figure S1

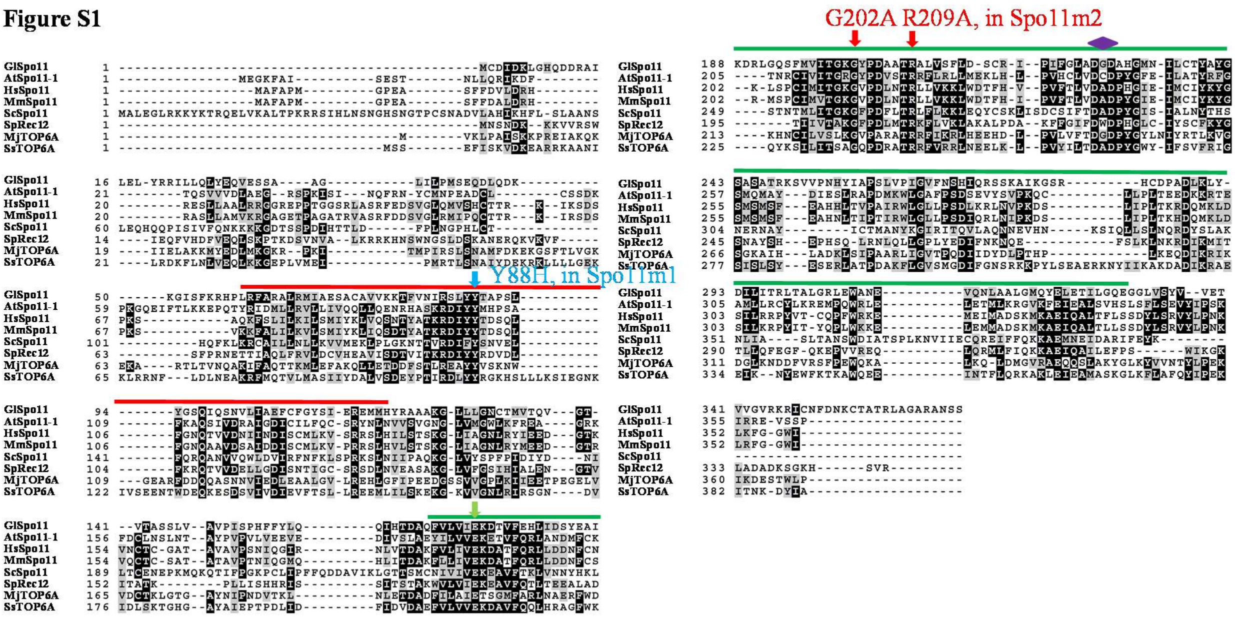

Fig. S1. Alignment of the TOP6A topoisomerases and Spo11. The TOP6A topoisomerases or Spo11 from different organisms, including *Methanocaldococcus jannaschii* (Mj), *Sulfolobus shibatae* (Ss), *Arabidopsis thaliana* (At), *Homo sapiens* (Hs), *Mus musculus* (Mm), *Schizosaccharomyces pombe* (Sp), *Saccharomyces cerevisiae* (Sc), and *Giardia lamblia* (Gl) are analyzed by ClustalW 1.83 with all default settings. GenBank accession numbers for MjTOP6A, SsTOP6A, AtSpo11-1, HsSpo11, MmSpo11, SpRec12 (Spo11), ScSpo11, and GlSpo11 are WP_010869868.1, WP_218258439.1, NP_187923.1, NP_937998.1, NP_001077429.1, NP_593479.1, AJV27957.1, and AAP35104.1, respectively. Black boxes, gray boxes and hyphens indicate identical amino acids, conserved amino acids and gaps in the respective proteins, respectively. The catalytic important Tyrosine 88 pointed by a blue arrow was mutated to produce Spo11m1. The conserved Glu and two Asp (DxD) in the Toprim domain is indicated by a green arrow and purple diamond, respectively. Two residues, Glu202 and Arg209 in the Toprim domain of *Giardia* Spo11, were mutated to produce Spo11m2.

Figure S2

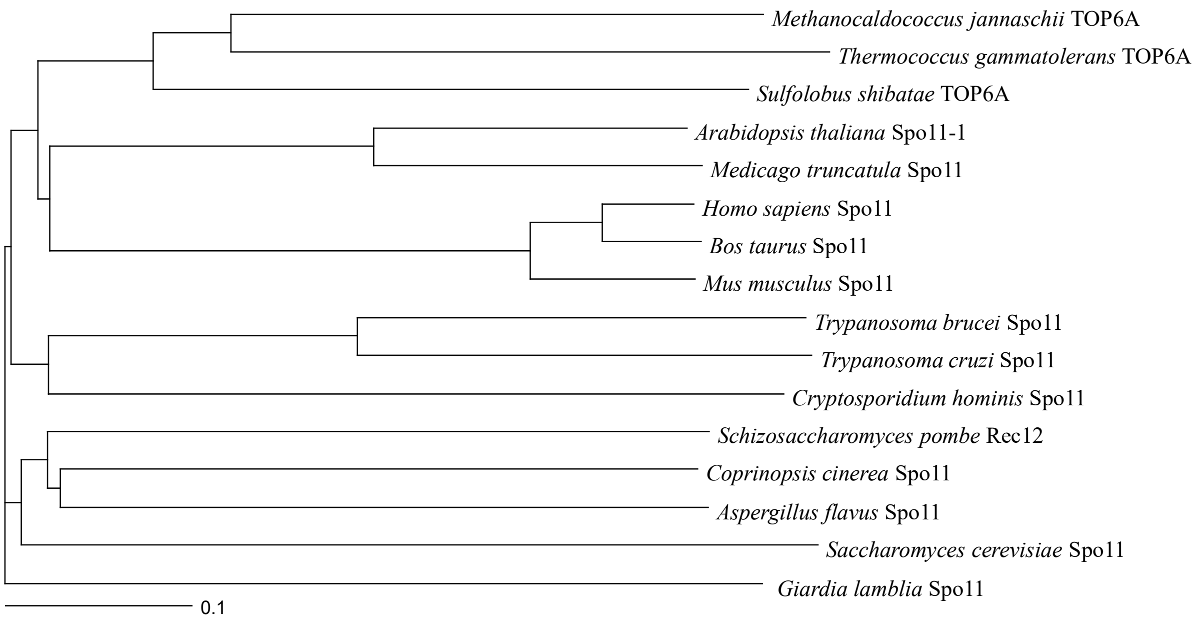
Methanocaldococcus jannaschii TOP6A
Thermococcus gammatolerans TOP6A
Sulfolobus shibatae TOP6A
Arabidopsis thaliana Spo11-1
Medicago truncatula Spo11
Homo sapiens Spo11
Bos taurus Spo11
Mus musculus Spo11
Trypanosoma brucei Spo11
Trypanosoma cruzi Spo11
Cryptosporidium hominis Spo11
Schizosaccharomyces pombe Rec12
Coprinopsis cinerea Spo11
Aspergillus flavus Spo11
Saccharomyces cerevisiae Spo11
Giardia lamblia Spo11
0.1

<u>Fig. S2.</u> Phylogenetic analysis of the TOP6A topoisomerases and Spo11. A neighbor-joining phylogenetic tree was obtained from alignment of TOP6A topoisomerases and Spo11 from various organisms, including *Methanocaldococcus jannaschii* (Mj), *Thermococcus gammatolerans* (Tg), *Sulfolobus shibatae* (Ss), *Arabidopsis thaliana* (At), *Medicago truncatula* (Mt), *Homo sapiens* (Hs), *Bos Taurus* (Bt), *Mus musculus* (Mm), *Trypanosoma brucei brucei* (Tb), *Trypanosoma cruzi* (Tc), *Cryptosporidium hominis* (Ch), *Schizosaccharomyces pombe* (Sp), *Coprinopsis cinerea* (Cc), *Aspergillus flavus* (Ao), *Saccharomyces cerevisiae* (Sc), and *Giardia lamblia* (Gl). GenBank accession numbers for MjTOP6A, TgTOP6A, SsTOP6A, AtSpo11-1, MtSpo11, HsSpo11, BtSpo11, MmSpo11, TbSpo11, TcSpo11, ChSpo11, SpRec12 (Spo11), CcSpo11, AfSpo11, ScSpo11, and GlSpo11 are WP_010869868.1, WP_015859039.1, WP_218258439.1, NP_187923.1, AET00713.2, NP_937998.1, NP_001180019.1, NP_001077429.1, XP_845046.1, EKF98601.1, XP_666249.1, NP_593479.1, AAF26720.1, QRD90412.1, AJV27957.1, and AAP35104.1, respectively. The bootstrap values determined from 1000 trees are not shown. Values are higher than 400. 0.1 substitution per site in the protein is indicated.

Figure S3

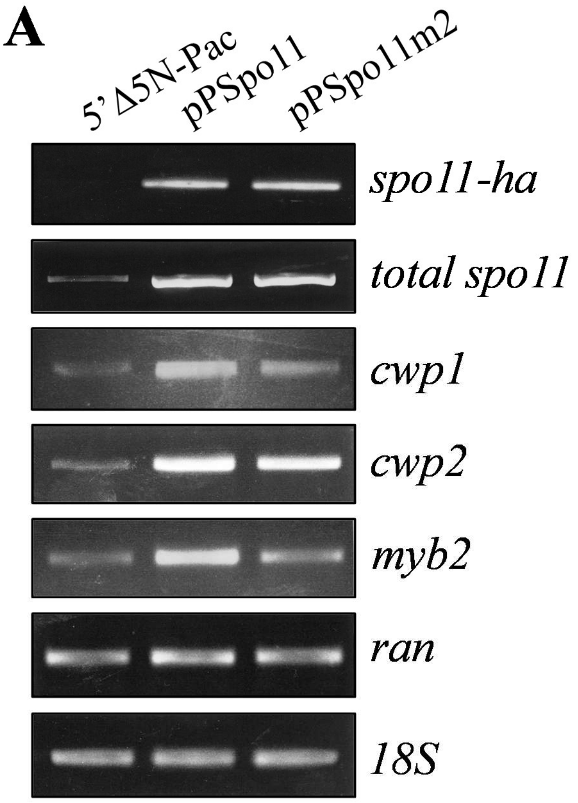
A
5' Δ5N-Pac
pPSpo11
pPSpo11m2
spo11-ha
total spo11
cwp1
cwp2
myb2
ran
18S

__Fig. S3__. **RT-PCR assays of transcript expression in the Spo11- and Spo11m2-expressing cell lines.** RNA was extracted from the 5’Δ5N-Pac, pPSpo11 and pPSpo11m2 stable transfectants cultured in growth medium. RT-PCR was performed using primers for genes indicated in the figure. As a control, similar levels of the 18S ribosomal RNA (18S) were detected.

Figure S4

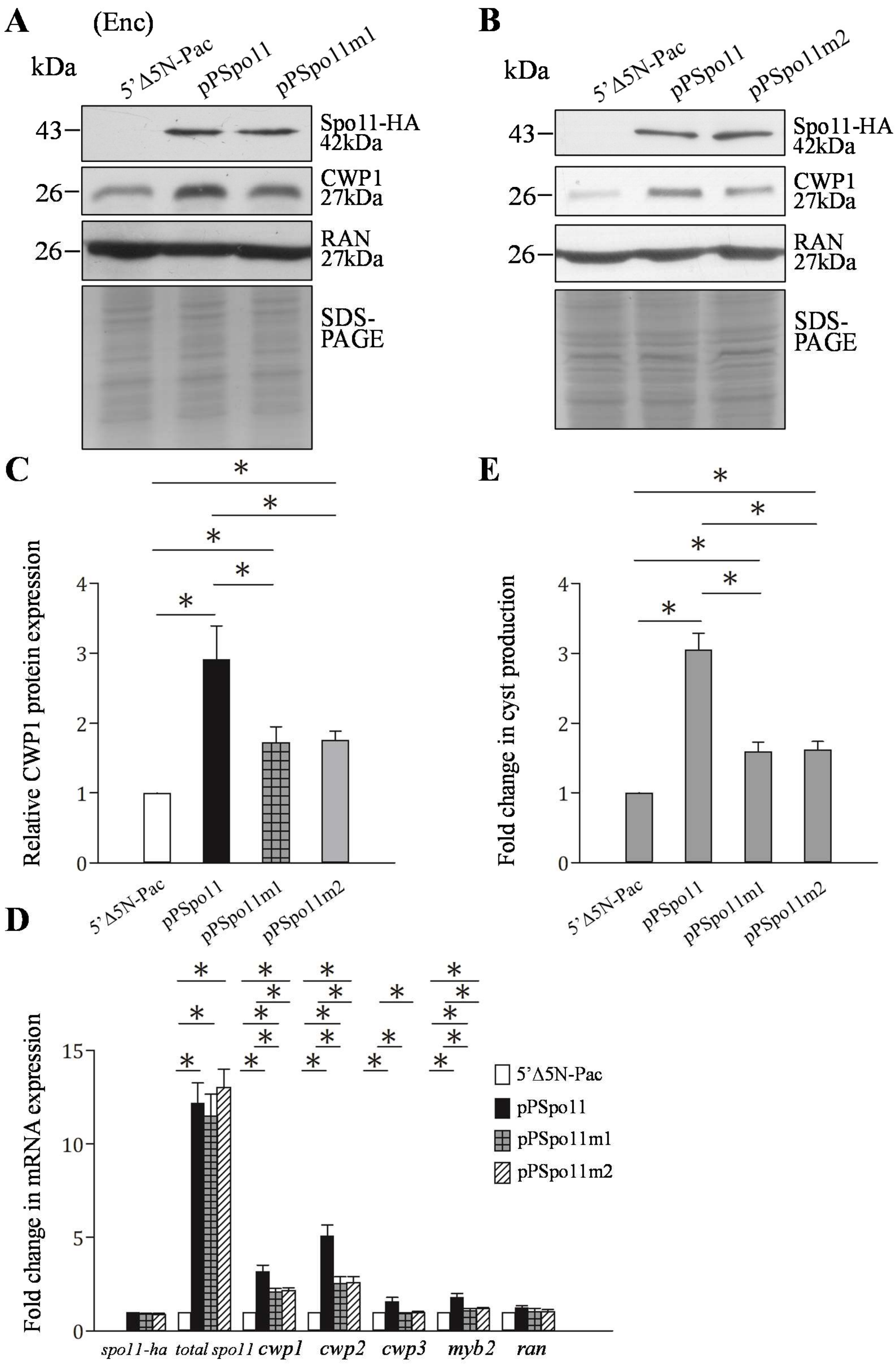
A
(Enc)
kDa
5'Δ5N-Pac
pPSpo11
pPSpo11m1
43
26
26
Spo11-HA
42kDa
CWP1
27kDa
RAN
27kDa
SDS-PAGE
B
kDa
5'Δ5N-Pac
pPSpo11
pPSpo11m2
43
26
26
Spo11-HA
42kDa
CWP1
27kDa
RAN
27kDa
SDS-PAGE
C
Relative CWP1 protein expression
0
1
2
3
4
5'Δ5N-Pac
pPSpo11
pPSpo11m1
pPSpo11m2
E
Fold change in cyst production
0
1
2
3
4
5'Δ5N-Pac
pPSpo11
pPSpo11m1
pPSpo11m2
D
Fold change in mRNA expression
0
5
10
15
spo11-ha
total spo11
cwp1
cwp2
cwp3
myb2
ran
5'Δ5N-Pac
pPSpo11
pPSpo11m1
pPSpo11m2

<u>Fig.S4.</u> Increased expression of the *cwp1-3* and *myb2* genes in the Spo11-expressing cell line during encystation. (A) Spo11 expression increased the CWP1 level during encystation. The 5'Δ5N-Pac, pPSpo11, and pPSpo11m2 stable transfectants were incubated in encystation medium for 24h and then subjected to SDS-PAGE and Western blot. The blot was probed by anti-HA, anti-CWP3, and anti-Ran antibodies, respectively. The intensity of bands from three Western blot assays was quantified as described in Fig. 3A. (B) Quantitative real-time PCR assays of transcript expression in the Spo11- and Spo11m2- expressing cell lines during encystation. Real-time PCR was performed using primers for genes indicated in the figure. The ratio of mRNA levels in the pPSpo11 or pPSpo11m2 cell line to levels in the 5'Δ5NPac cell line was quantified as described in Fig. 3C. (D) Expression of Spo11 induced cyst generation during encystation. Cyst number was counted from the 5'Δ5N-Pac, pPSpo11, and pPSpo11m2 stable transfectants cultured in encystation medium as described in "Materials and Methods" and Fig. 3D.

Figure S5

A

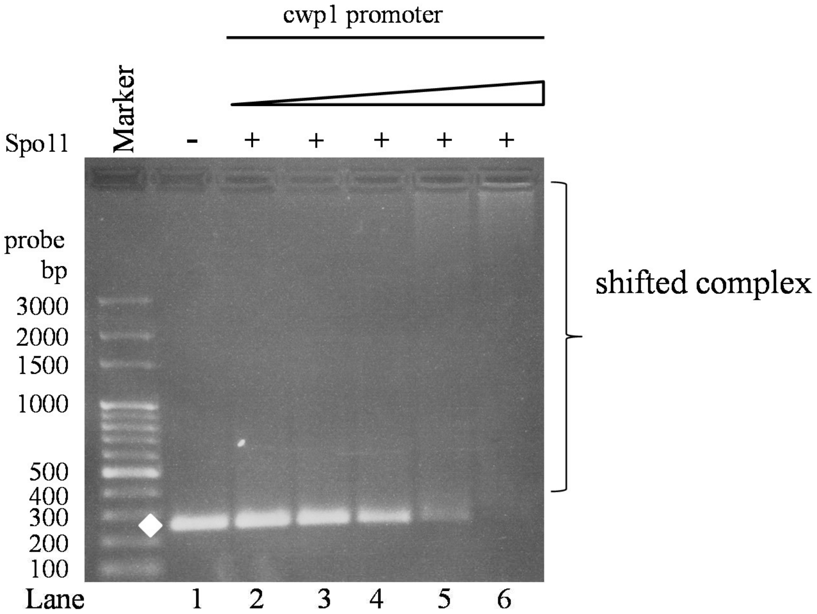

cwp1 promoter
Marker
Spo11
-
+
+
+
+
+
probe
bp
3000
2000
1500
1000
500
400
300
200
100
shifted complex
Lane
1
2
3
4
5
6

B

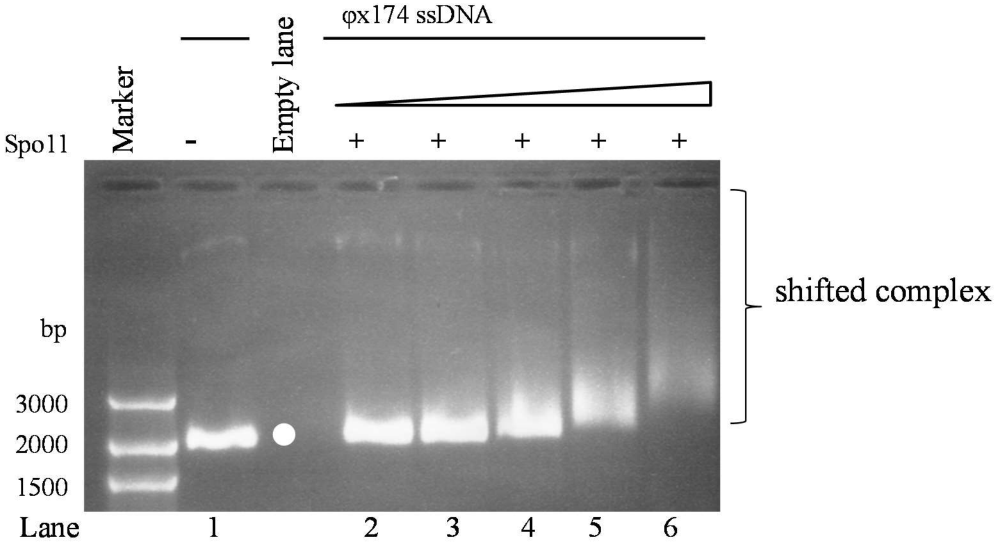

φx174 ssDNA
Marker
Empty lane
Spo11
-
+
+
+
+
+
bp
3000
2000
1500
shifted complex
Lane
1
2
3
4
5
6

Fig. S5. Spo11 can bind to *cwp1* promoter and ssDNA. (A) Detection of DNA binding ability of Spo11 with *cwp1* promoter probe. Purified Spo11 and the linear *cwp1* promoter probe were used in electrophoretic mobility shift assays. Binding reaction mixtures contained the components indicated above the lanes. The probe (300ng/rxn) was incubated with 26, 53, 105, 210, and 420ng purified Spo11 (lanes 2 to 6, respectively). The white diamond indicates linear DNA. (B) Detection of DNA binding ability of Spo11 with ssDNA probe. Purified Spo11 and the φx174 ssDNA probe were used in electrophoretic mobility shift assays. Binding reaction mixtures contained the components indicated above the lanes. The probe (300ng/rxn) was incubated with 26, 53, 105, 210, and 420ng purified Spo11 (lanes 2 to 6, respectively). The white circle indicates ssDNA.

Figure S6

ATGGGCACCGAGTACAAGCCCACGGTGCGCCTCGCCACCCGCGACGACGTCCCCCGGGCCG
TACGCACCCTCGCCGCCGCGTTCGCCGACTACCCCGCCACGCGCCACACCGTCGACCCGGA
CCGCCACATCGAGCGGGTCACCGAGCTGCAAGAACTCTTCCTCACGCGCGTCGGGCTCGAC
ATCGGCAAGGTGTGGGTCGCGGACGACGGCGCCGCGGTGGCGGTCTGGACCACGCCGGAGA
GCGTCGAAGCGGGGGCGGTGTTCGCCGAGATCGGCCCGCGCATGGCCGAGTTGAGCGGTTC
CCGGCTGGCCGCGCAGCAACAGATGGAAGGCCTCCTGGCGCCGCACCGGCCCAAGGAGCCC
GCGTGGTTCCTGGCCACCGTCGGCGTCTCGCCCGACCACCAGGGCAAGGGTCTGGGCAGCG
CCGTCGTGCTCCCCGGAGTGGAGGCGGCCGAGCGCGCCGGGGTGCCCGCCTTCCTGGAGAC
CTCCGCGCCCCGCAACCTCCCCTTCTACGAGCGGCTCGGCTTCACCGTCACCGCCGACGTC
GAGTGCCCG**AAGGACCGCGCGACCTGG**TGCATGACCCGCAAGCCCGGTGCCCTCGAGTGA
cggtggtgcttggtcagatttgtgcaattgggaactctgcaagtggcgtgtttaacgacct
tttgtcagggattaacgtctctgtacccttcttgcagtctatgctcttctacgggctgctt
ctatttttatgggtactgccttccgttcacaagctattcgtccaccgtgcccgcgatgtag
gctttttcttctatctgggatcctagatatcacagcaaacagccttgccataatgtcatt
tgtttacacatctgtcggcgccgttctactcattctttgtctctctacgcccttctccatg
atcctctcgttgatcattacgaaagcgcgcttctcgtggatgcaagtgatgttttcctgct
tcgctacggggttcgcgatattgtttgtcattcttgacactatgggcgacgagtcaaaaca
cagggtgcttggagatttgcttgctgtagcctcagcctttatctacgggctcacatccgtg
ataaatgagttcgtcataggctcatatacacctgtccaattccttgccaggctcagcatcg
gcgccttttctctcgcgcttattctgtttctctgtttcgaggcagacaacatccagatttt
ggcgacccttcggccgtggtggtatatcataggatatctcgtcagcctcgtggtgatgtac
agtgttctcccgttagtgattaagtacggcggtgcagttgttttcaatatcagtctcataa
gttgtaatgtttatggaatgtttgcgtccttgatcatattcaagtac**aaatatatcccttg**
gattgca

Fig. S6. Replacement of the *spo11* gene with the *pac* gene in the Spo11td cell line confirmed by PCR and sequencing. Genomic DNA was isolated from the Spo11td and control cell lines cultured in growth medium. PCR was performed using primers specific for *pac* (PCR2 in Fig. 7A), which are PCR2F for bold region 1 and PCR2R for bold region 2, to verify the integration of the *pac* gene into the correct region in genomic DNA. The sequence results obtained from the PCR2 product are shown as underlined letters. Capital letters indicate the coding sequence for the *pac* gene, which starts at ATG and stops at TGA. This indicates the replacement of the *spo11* gene with the *pac* gene. The region used to clone the *spo11* 3' region into the pSpo11td plasmid for HR is shown in red, which is also between the sequence of spo113XF and spo113KR. The underlined and lower case letters, which are downstream and outside of the red region of spo113XF and spo113KR, indicate that HR occurred in the sequence of spo11 3' region and that the *pac* gene was integrated in the genomic DNA.

Figure S7

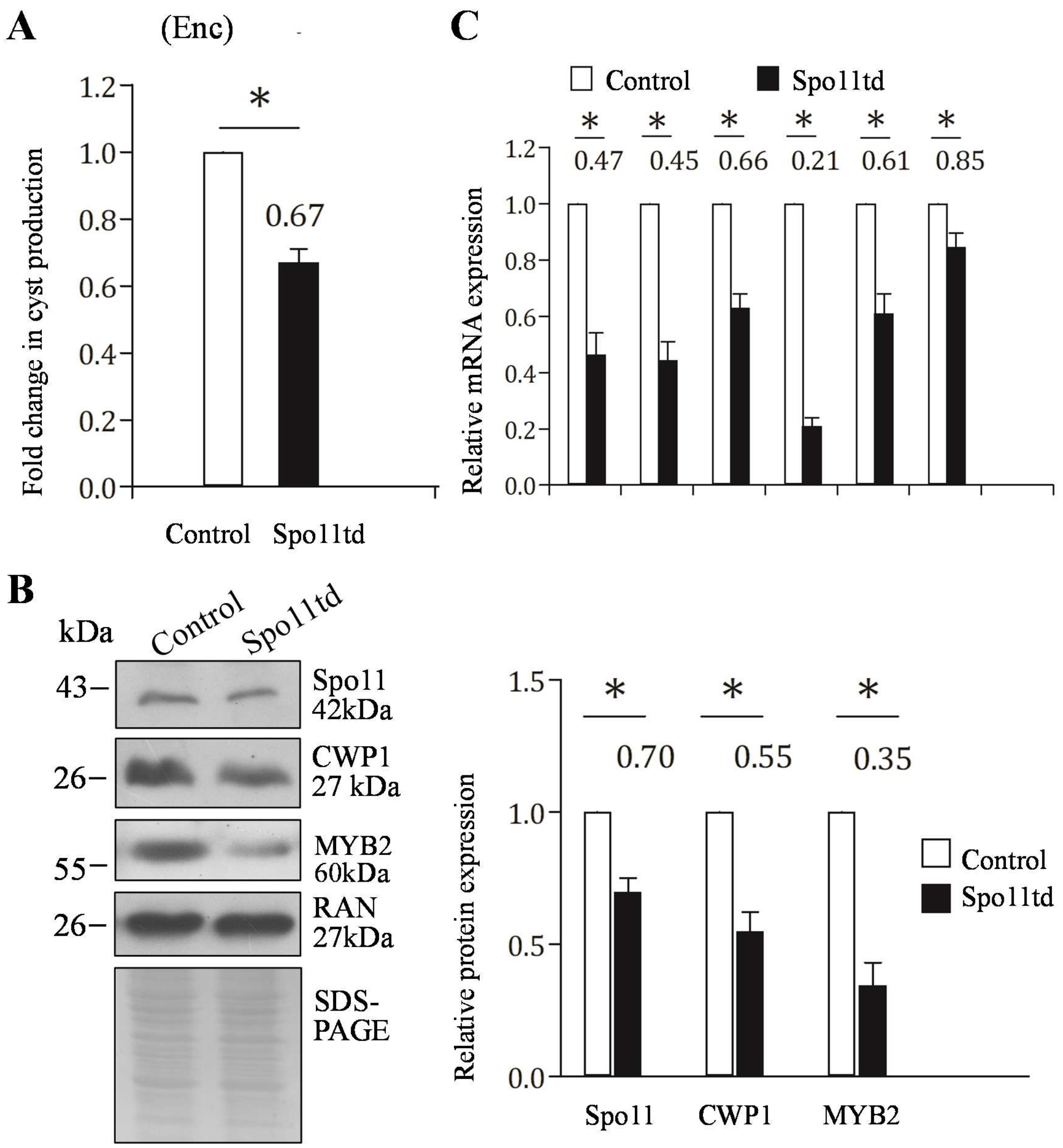
A
(Enc)
Fold change in cyst production
1.2
1.0
0.8
0.6
0.4
0.2
0.0
*
0.67
Control
Spo11td
C
Control
Spo11td
Relative mRNA expression
*
0.47
0.45
0.66
0.21
0.61
0.85
B
kDa
Control
Spo11td
43
26
55
26
Spo11 42kDa
CWP1 27 kDa
MYB2 60kDa
RAN 27kDa
SDS-PAGE
Relative protein expression
1.5
1.0
0.5
0.0
0.70
0.55
0.35
Control
Spo11td
Spo11
CWP1
MYB2

<u>Fig. S7.</u> Decrease in expression of *cwp1-3*, and *myb2* by targeted disruption of the *spo11* gene during encystation. (A) Cyst generation decreased by targeted disruption of the *spo11* gene in the Spo11td cell line during encystation. The control and Spo11td cell lines were cultured in encystation medium for 24h (Enc) and then subjected to cyst count as described under "Materials and Methods" and Fig. 3D. (B) Targeted disruption of the *spo11* gene decreased the CWP1 and MYB2 levels in the Spo11td cell line during encystation. The control and Spo11td cell lines were cultured in encystation medium for 24h and then subjected to SDS-PAGE and Western blot analysis as described in Fig. 3A. The blot was probed with anti-Spo11, anti-CWP1, anti-MYB2, and anti-RAN antibodies, respectively. The intensity of bands from three Western blot assays was quantified as described in Fig. 3A. (C) Decrease in expression of *cwp1-3*, and *myb2* by targeted disruption of the *spo11* gene in the Spo11td cell line during encystation. The control and Spo11td cell lines were cultured in encystation medium for 24h and then subjected to quantitative real-time RT-PCR analysis using primers specific for *spo11*, *cwp1*, *cwp2*, *cwp3*, *myb2*, *ran*, and 18S ribosomal RNA genes, respectively, as described in Fig. 2A.

Figure S8

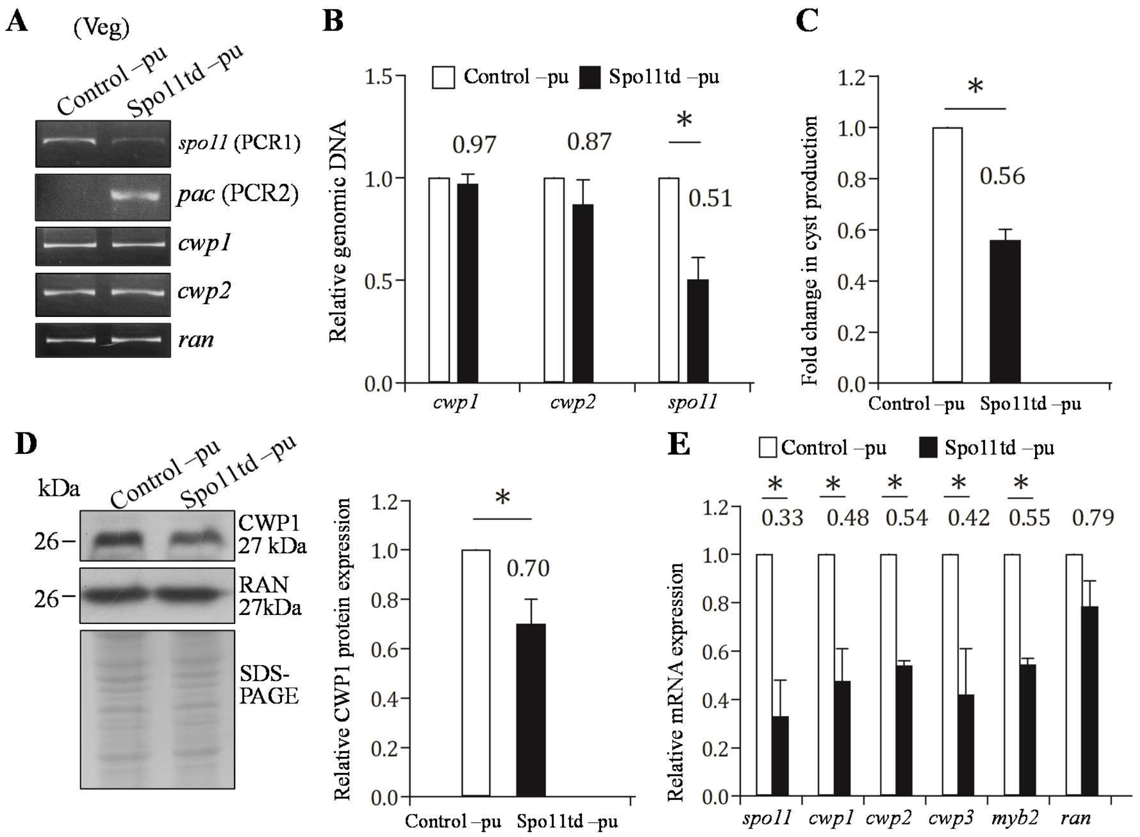
A
(Veg)
Control -pu
Spo11td -pu
spo11 (PCR1)
pac (PCR2)
cwp1
cwp2
ran
B
Control -pu
Spo11td -pu
Relative genomic DNA
0.97
0.87
0.51
*
cwp1
cwp2
spo11
C
Fold change in cyst production
*
0.56
Control -pu
Spo11td -pu
D
kDa
Control -pu
Spo11td -pu
26
CWP1
27 kDa
26
RAN
27kDa
SDS-
PAGE
Relative CWP1 protein expression
*
0.70
Control -pu
Spo11td -pu
E
Control -pu
Spo11td -pu
Relative mRNA expression
0.33
0.48
0.54
0.42
0.55
0.79
spo11
cwp1
cwp2
cwp3
myb2
ran

Fig. S8. Decrease in expression of *cwp1-3* and *myb2* by targeted disruption of the *spo11* gene after the removal of puromycin during vegetative growth. (A) Partial replacement of the *spo11* gene with the *pac* gene in the Spo11 –pu cell line confirmed by PCR. Puromycin was removed from the Spo11td and control cell lines to obtain the Spo11td –pu and control –pu cell lines, respectively. Genomic DNA was isolated from the Spo11td –pu and control -pu cell lines cultured in growth medium (vegetative growth, Veg). PCR was performed using primers specific for *spo11* (PCR1), *pac* (PCR2), *cwp1*, *cwp2*, and *ran* genes, respectively, as described in Fig. 8B. (B) Partial disruption of the *spo11* gene in the Spo11td -pu cell line confirmed by real-time PCR. Real-time PCR was performed using genomic DNA and primers specific for *spo11*, *cwp1*, *cwp2*, and *ran* genes, respectively, as described in Fig. 8C. (C) Cyst generation decreased by targeted disruption of the *spo11* gene in the Spo11td –pu cell line during vegetative growth. The control –pu and Spo11td –pu cell lines were cultured in growth medium and then subjected to cyst count as described under "Materials and Methods" and Fig. 3D. (D) Targeted disruption of the *spo11* gene decreased the CWP1 level in the Spo11td –pu cell line during vegetative growth. The control –pu and Spo11td –pu cell lines were cultured in growth medium and then subjected to SDSPAGE and Western blot analysis as described in Fig. 3A. The blot was probed with anti-CWP1, and anti-RAN antibodies, respectively. The intensity of bands from three Western blot assays was quantified as described in Fig. 3A. (E) Decrease in expression of *cwp1-3* and *myb2* by targeted disruption of the *spo11* gene in the Spo11td –pu cell line during vegetative growth. The control –pu and Spo11td –pu cell lines were cultured in growth medium and then subjected to quantitative real-time RTPCR analysis using primers specific for *spo11*, *cwp1*, *cwp2*, *cwp3*, *myb2*, *ran*, and 18S ribosomal RNA genes, respectively, as described in Fig. 2A.

Figure S9

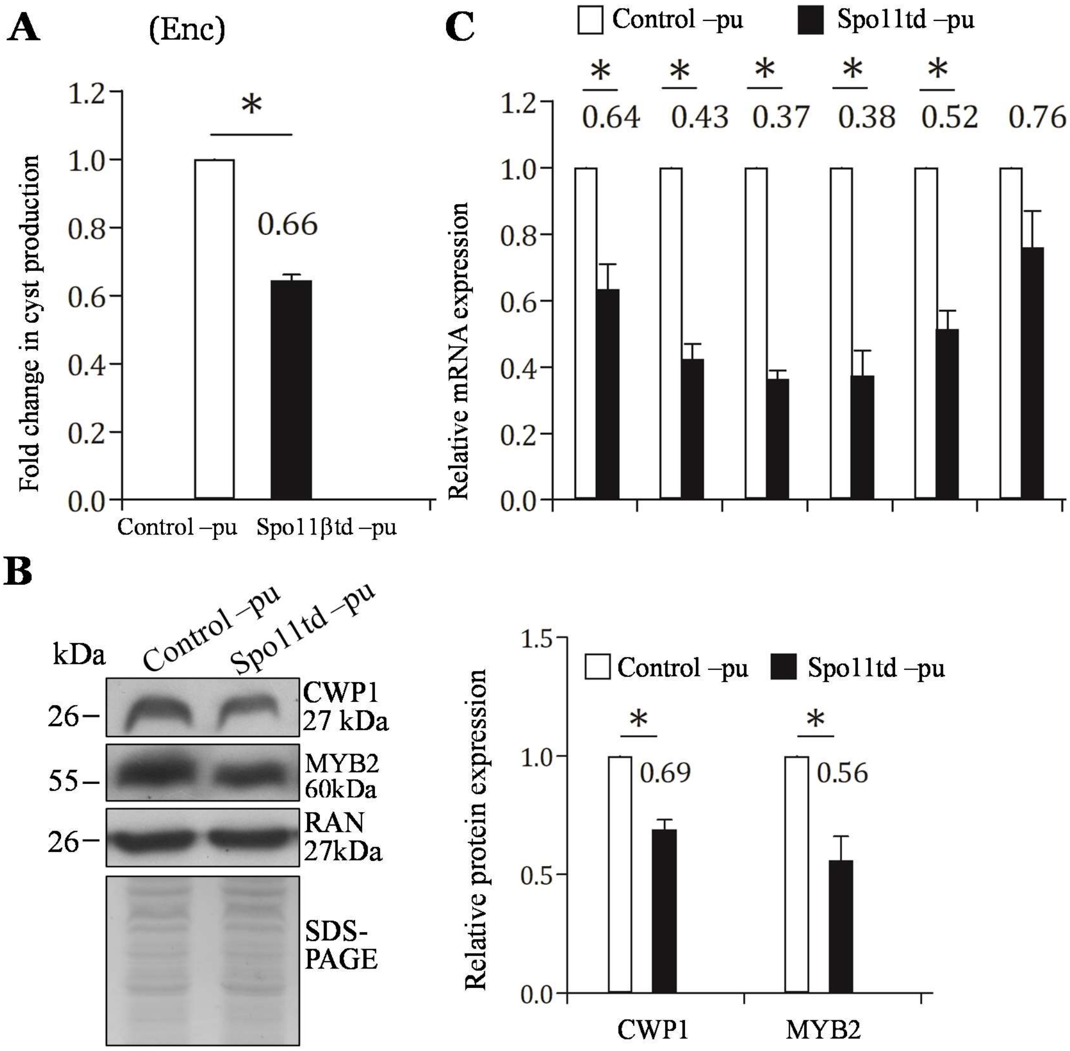

D

-200
GGATGGTTAGTAACGCTTCCCCGTTCCTTAGTGTAGTGCATATGTTGTCGTCTGTGAGGATGCA

TGCTGGATTGAGGCTTTGCACCCATCGTGCTAGTAGCTGCTGTTCA***CTGACT***TGTCTGTTACAA

GAGACTCTACAGGATACTTTCAGGTTTTCTACAGC**AAATTTGTATTGATTAAT**CAACAATCTGG

-1
TGTCTGAC

Fig. S9. Decrease in expression of *cwp1* and *cwp2* by targeted disruption of the *spo11* gene after the removal of puromycin during encystation. (A) Cyst generation decreased by targeted disruption of the *spo11* gene in the Spo11td –pu cell line during encystation. The control –pu and Spo11td –pu cell lines were cultured in encystation medium for 24h (Enc) and then subjected to cyst count as described under "Materials and Methods", as described in Fig. 3D. (B) Targeted disruption of the *spo11* gene decreased the CWP1 level in the Spo11td –pu cell line during encystation. The control –pu and Spo11td –pu cell lines were cultured in encystation medium for 24h and then subjected to SDS-PAGE and Western blot analysis as described in Fig. 3A. The blot was probed with anti-CWP1, and anti-RAN antibodies, respectively. The intensity of bands from three Western blot assays was quantified as described in Fig. 3A. (C) Decrease in expression of *cwp1 -3* and *myb2* by targeted disruption of the *spo11* gene in the Spo11td –pu cell line during encystation. The control –pu and Spo11td –pu cell lines were cultured in encystation medium for 24h and then subjected to quantitative real-time RT-PCR analysis using primers specific for *spo11*, *cwp1*, *cwp2*, *cwp3, myb2, ran*, and 18S ribosomal RNA genes, respectively, as described in Fig. 2A. (D) The WRKY and MYB2 binding sites in the *Spo11* promoter. The 200-bp 5' untranslated region of the *spo11* promoter is shown. The AT-rich promoter element is in bold. The underlined sequence, CTGACT and CTACAG, is the WRKY and MYB2 binding sequences, respectively.

Table S2. Oligonucleotides used for construction of plasmids and PCR.

Name	Sequence (5'--->3')
spo11F (PCR1F)	CACCATGTGCGATATTGATAAG
spo11R (PCR1R)	GGACGAATTTGCGCGAGC
HAR	AGCGTAATCTGGAACATCGTATGGGTA
cwp1F	ATGATGCTCGCTCTCCTT
cwp1R	TCAAGGCGGGGTGAGGCA
cwp2F	ATGATCGCAGCCCTTGTTCTA
cwp2R	CCTTCTGCGGACAATAGGCTT
cwp3F	ATGTTTTCTCTGCTTCTTCT
cwp3R	TCTGTAGTAGGGCGGCTGTA
myb2F	ATGTTACCGGTACCTTCTCAGC
myb2R	GGGTAGCTTCTCACGGGGAAG
ranF	ATGTCTGACCCAATCAGC
ranR	TCAATCATCGTCGGGAAG
spo11realF	CGCAGAATTTTGCTTCGGATA
spo11realR	GCAGCTGCCCGATAATGC
cwp1realF	AACGCTCTCACAGGCTCCAT
cwp1realR	AGGTGGAGCTCCTTGAGAAATTG
cwp2realF	TAGGCTGCTTCCCACTTTTGAG
cwp2realR	CGGGCCCGCAAGGT
cwp3realF	GCAAATTGGATGCCAAACAA
cwp3realR	GACTCCGATCCAGTCGCAGTA
myb2realF	TCCCTAATGACGCCAAACG
myb2realR	AGCACGCAGAGGCCAAGT
ranrealF	TCGTCCTCGTCGGAAACAA
ranrealR	AACTGTCTGGGTGCGGATCT
18SrealF	AAGACCGCCTCTGTCAATCAA
18SrealR	GTTTACGGCCGGGAATACG
spo11NF	GGCCGGCTAGCTATTGAAGAACGACATCAAGA
spo11MR	GGCCGACGCGTGGACGAATTTGCGCGAGCACC

spo11m1F	AACATTCGCAGTTTGTATCATACTGCTCCTTCGCTATAT
spo11m1R	ATATAGCGAAGGAGCAGTATGATACAAACTGCGAATGTT
spo11m2F	GTGATCACTGGCAAAGCATACCCCGATGCGGCGACAGCCGCCCTGGTTTCTTTT
spo11m2R	AAAAGAAACCAGGGCGGCTGTCGCCGCATCGGGGTATGCTTTGCCAGTGATCAC
spo115HF	GGCCGAAGCTTTGTCATTTGCAATTGTGTTAG
spo115NR	GGCCGCCATGGGTCAGACACCAGATTGTTGAT
spo113XF	GGCCGCTCGAGTGACGGTGGTGCTTGGTCAGATTT
spo113KR	GGCCGGGTACCCCGTACTTAATCACTAACGGG
spo11-guide	GAGAGCGGGTACCCTAGCTTATTGAAAAAGCGAGAGGCCATTGAGATGACTCGCCTGATTGCAATAGCAAACAGTGTCTATAGTCTAATTGTGGACAACAGAGGGCTTATTGCAACGTTGATGACCAAGTTCAACAAGGGCGTCGCCTCCTATGAGCAGGTCATTCGGAATTTCGACGACCGGTAGCGTCCCCAGAGTAAACCATTTTAAATTGAAATAGGCGGTTGGAAATAAAAGCGCGCC**TACACTGCTCCTTCGCTATA**GTTTTAGAGCTAGAAATAGCAAGTTAAAATAAGGCTAGTCCGTTATCAACTTGAAAAAGTGGCACCGAGTCGGTGCTTTTTTTGAATTCGAGAGCG, underlined region is U6 promoter, bold region is for annealing, underlined and bold region is upstream 3nt of PAM, other region is scaffold RNA
PCR2F	AAGGACCGCGCGACCTGG
PCR2R	TGGACAAAGAAAGTCTCGGGC
spo115F	TAGTGTAGTGCATATGTTGTC
spo115R	GTAGAGTCTCTTGTAACAGAC
18S5F	CCAAAAAAGTGTGGTGCAGG
18S5R	GCCGGGCGCGGGCGCCGCGG
cwp15F	CAACGGCTTACTAAATCATTCTCTTG
cwp15R	TTCTGTGTTTCTTGATCTGAGAGTTGT
cwp25F	CACTTTGATGAGAGCATGGG
cwp25R	TTAGTTCATATCTTAAGTTA
cwp35F	TGGGGGAGATAGGAGAATAC
cwp35R	ATCAGTAGTAACTTATTTTTTGGGAAAGAC
myb25F	TGGCTATGTATTTTTTCTTCTTCTACAGCT
myb25R	TAGCAGTACAGAGTAATTATTATTTTAGTA
U65F	TTGAGATGACTCGCCTGATTG
U65R	GAAATTCCGAATGACCTGCTC
